# Development of a time-series shotgun metagenomics database for monitoring microbial communities at the Pacific coast of Japan

**DOI:** 10.1038/s41598-021-91615-3

**Published:** 2021-06-09

**Authors:** Kazutoshi Yoshitake, Gaku Kimura, Tomoko Sakami, Tsuyoshi Watanabe, Yukiko Taniuchi, Shigeho Kakehi, Akira Kuwata, Haruyo Yamaguchi, Takafumi Kataoka, Masanobu Kawachi, Kazuho Ikeo, Engkong Tan, Yoji Igarashi, Masafumi Ohtsubo, Shugo Watabe, Yutaka Suzuki, Shuichi Asakawa, Sonoko Ishino, Kosuke Tashiro, Yoshizumi Ishino, Takanori Kobayashi, Katsuhiko Mineta, Takashi Gojobori

**Affiliations:** 1 Laboratory of Aquatic Molecular Biology and Biotechnology, Graduate School of Agricultural and Life Sciences, The University of Tokyo, Yayoi, Bunkyo, Tokyo Japan; 2 Japan Software Management Co., Ltd., Kinko-cho, Yokohama, Kanagawa Japan; 3 Japan Fisheries Research and Education Agency, Shinurashima, Kanagawa, Yokohama, Kanagawa Japan; 4 Biodiversity Division, National Institute for Environmental Studies, 16-2 Onogawa, Tsukuba, Ibaraki Japan; 5 Faculty of Marine Science and Technology, Fukui Prefectural University, 1-1 Gakuen-cho, Obama, Fukui Japan; 6 Department of Genetics, SOKENDAI, Mishima, Japan; 7 National Institute of Genetics, Mishima, Japan; 8 Preeminent Medical Photonics Education & Research Center, Hamamatsu University School of Medicine, 1-20-1 Handayama, Higashi-ku, Hamamatsu, Shizuoka Japan; 9 School of Marine Biosciences, Kitasato University, Sagamihara, Kanagawa Japan; 10 Department of Computational Biology and Medical Sciences, Graduate School of Frontier Sciences, The University of Tokyo, 5-1-5 Kashiwanoha, Kashiwa, Chiba 277-8561 Japan; 11 Department of Bioscience and Biotechnology, Graduate School of Bioresource and Bioenvironmental Sciences, Kyushu University, Fukuoka, Japan; 12 Computational Bioscience Research Center, King Abdullah University of Science and Technology, Thuwal, Saudi Arabia

**Keywords:** Genetic databases, Microbial ecology

## Abstract

Although numerous metagenome, amplicon sequencing-based studies have been conducted to date to characterize marine microbial communities, relatively few have employed full metagenome shotgun sequencing to obtain a broader picture of the functional features of these marine microbial communities. Moreover, most of these studies only performed sporadic sampling, which is insufficient to understand an ecosystem comprehensively. In this study, we regularly conducted seawater sampling along the northeastern Pacific coast of Japan between March 2012 and May 2016. We collected 213 seawater samples and prepared size-based fractions to generate 454 subsets of samples for shotgun metagenome sequencing and analysis. We also determined the sequences of 16S rRNA (n = 111) and 18S rRNA (n = 47) gene amplicons from smaller sample subsets. We thereafter developed the Ocean Monitoring Database for time-series metagenomic data (http://marine-meta.healthscience.sci.waseda.ac.jp/omd/), which provides a three-dimensional bird’s-eye view of the data. This database includes results of digital DNA chip analysis, a novel method for estimating ocean characteristics such as water temperature from metagenomic data. Furthermore, we developed a novel classification method that includes more information about viruses than that acquired using BLAST. We further report the discovery of a large number of previously overlooked (TAG)n repeat sequences in the genomes of marine microbes. We predict that the availability of this time-series database will lead to major discoveries in marine microbiome research.

## Introduction

With the advent of next-generation DNA sequencing, advanced metagenomic studies have been conducted worldwide, starting with the first shotgun metagenomic sequence analysis of the waters of the Sargasso Sea by Venter et al.^1^. Metagenome sequencing approaches can be broadly categorized into amplicon and shotgun metagenome sequencing. The former refers to the sequencing of a partial genomic region, such as an rRNA gene, previously amplified by polymerase chain reaction (PCR). In contrast, shotgun metagenomic sequencing allows the analysis of whole DNA preparation^2^. Shotgun metagenome sequencing is advantageous because it comprehensively detects organisms without PCR bias while targeting all sequences, including those of novel genes^3^. However, this approach is expensive and requires complex data analysis and assembly, which likely explains the fewer number of studies available on shotgun-sequenced metagenomes than those based on amplicon sequencing. However, recently developed software for the analysis of shotgun metagenomes by constructing metagenome-assembled genomes (MAGs) are available^4,5^. Consequently, the number of studies focusing on shotgun metagenomes has increased over the past decade.

Amplicon metagenomic databases include MetaMetaDB^6^, The European Bioinformatics Institute (EBI) metagenome database MGnify^7^, and MicrobeDB.jp (https://microbedb.jp/). There are also several shotgun metagenomic databases including MG-RAST^8^, IMG^9^, MGnify^7^and Ocean Gene Atlas^10^ for the purpose of searching and analyzing assembled shotgun metagenomic data. However, the shotgun metagenomic data in these databases is somewhat limited because the data were collected sporadically at different locations. Thus, these databases do not include long-term time-series data sampled at fixed locations. Long-term monitoring at fixed locations is important because marine amplicon metagenomic studies have shown that microbial communities can change significantly between seasons^11–14^. Few studies have reported time-series shotgun data. For instance, Viller et al.^8^ conducted a 2-year metagenomic time-series study at the ALOHA and BATS stations. However, a large-scale time-series shotgun metagenomic database has not yet been developed. Nevertheless, the time-series shotgun metagenome is an expanding field of research because the cost of sequencing is continuously decreasing.

To help filling the current gap in our knowledge of the structure and function of marine microbiomes, we routinely collected samples in the northeastern Pacific coastal region of Japan (Sendai Bay, Ofunato Bay, and Akkeshi off Hokkaido [A-line]) between 2012 and 2016. This region is rich in nutrients, such as nitrogen and phosphorus, vital for the growth of phytoplankton. With the seasonality of specific environmental variables such as nutrients and the availability of sunlight, certain biological groups follow seasonal patterns. In particular, large-scale phytoplankton blooms occur during spring in the northeastern coastal region of Japan in the western North Pacific^15^. These blooms supply food for the fish community, making this region rich in marine resources and one of the world’s largest fishing grounds.

The initiation and termination of blooms are typically monitored by observing the plankton community in seawater samples using microscopy. However, comprehensive analyses of small viruses, bacteria, and large phytoplankton have not been conducted, and the dynamics of the microbial community during the initiation and termination of blooms is unknown. Therefore, we conducted this study to determine changes in the relevant biota and identify the mechanisms causing these blooms. Our results will help determine microbial community dynamics which drive ecosystem functioning in this important fishing region. Among our study sites, Sendai Bay and Ofunato Bay are relatively easy to monitor due to their proximity to land. The A-line is a fixed observation transect that crosses the Oyashio current area into the Kuroshio–Oyashio transitional region. The A-line has been monitored for water temperature, salinity, chemical composition, phytoplankton, and zooplankton since 1987 (http://tnfri.fra.affrc.go.jp/seika/a-line/a-line_data.html). We, therefore, selected the A-line to compare the environmental conditions of past blooms with our observations of Sendai Bay and Ofunato Bay.

For this purpose, we performed sampling at least once every month in the northeastern marine region to construct a database to provide a more in-depth understanding of temporal marine metagenomics. We accumulated shotgun metagenomic data of seawater and analyzed the fluctuation patterns of the assembled contigs of the microbial communities. Furthermore, we developed the Ocean Monitoring Database (http://marine-meta.healthscience.sci.waseda.ac.jp/omd/) to display the time-series shotgun metagenomic data. Using this database, users can browse species composition over time using a two- or three-dimensional (3D) graphical view. Additionally, users can search for assembled genes and their abundance patterns according to nucleotide sequence homologies. This database will serve to enhance our understanding of the temporal dynamics in other oceans.

## Results and discussion

### Database construction

#### Collection of metagenomic data

**Figure 1:**
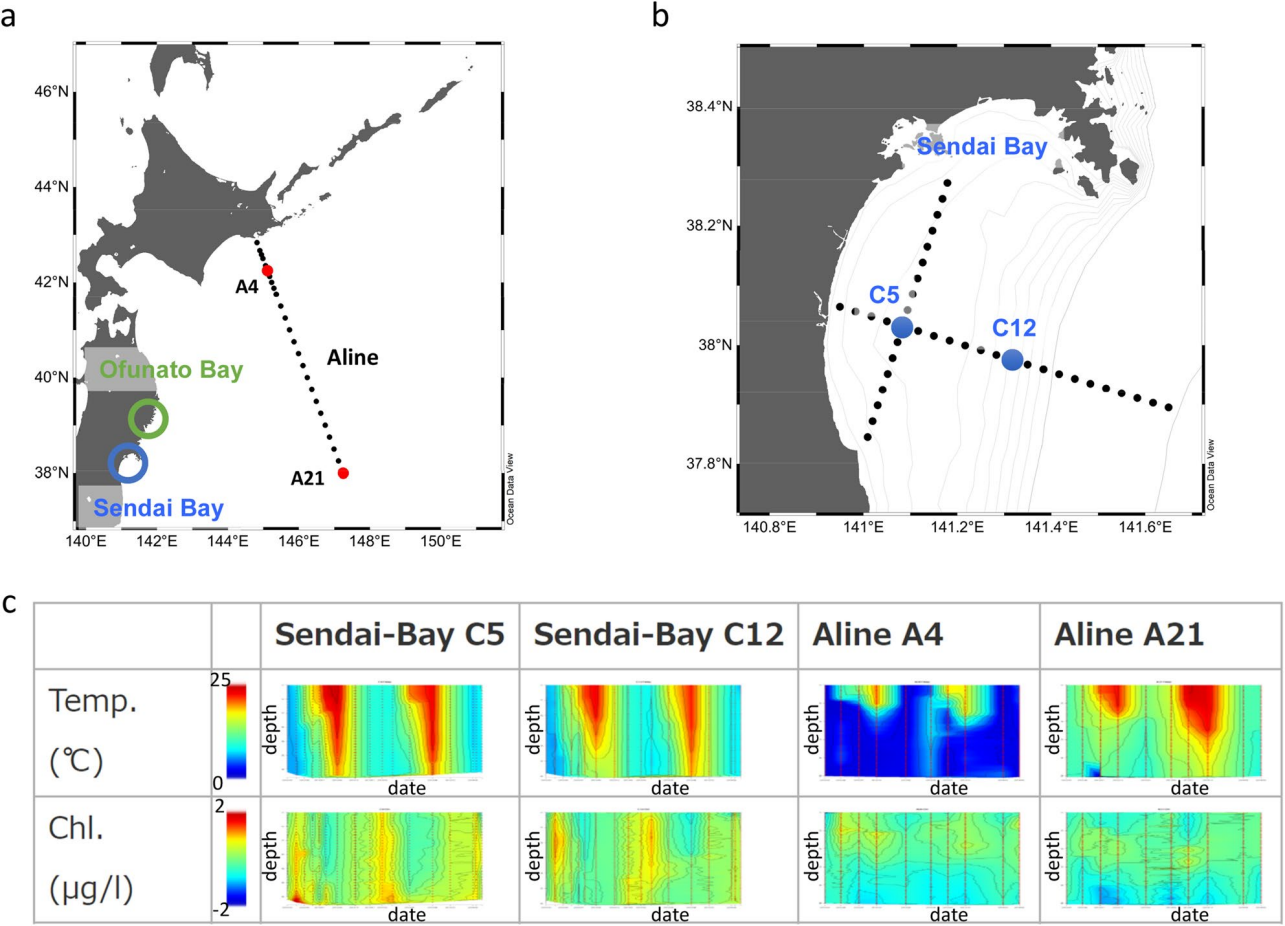
Location, changes in water temperature, and changes in chlorophyll-a (Chl-a) concentrations at the sampling points. (**a**) Sampling points along the Pacific coast of northeastern Japan (Sendai Bay, Ofunato Bay, and A-line). (**b**) Sampling points C5 and C12 in Sendai Bay. The map was generated using Ocean Data View (https://odv.awi.de) with data imported from the NOAA server (accessed on 22 February 2021). (**c**) Changes in water temperature and chlorophyll concentrations at Sendai Bay and A-line sampling points. Red circles indicate the depth of the sampled water. X-axis: dates from 2012 to 2014. Y-axis: water depth from the surface.

Between March 2012 and May 2016, seawater samples were collected from five different locations around Sendai Bay, Ofunato Bay, and A-line. Samples (N = 142) were collected from the surface and the subsurface chlorophyll-a maximum (SCM) layers (Fig. 1). DNA was extracted from microorganisms trapped in filters (pore sizes, 0.2, 0.8, 5, 20, and 100 μm). Shotgun metagenomic sequence data (N = 454) were acquired (Supplementary Information 1), comprising 3.57 × 10^9^ reads and 3.56 × 10^11^ bases in total (Supplementary Information 2). 16S rRNA gene sequences in the 0.2-μm fraction were subjected to PCR using universal primers to obtain 111 amplicon metagenomic sequences (6.92 × 10^6^ reads, 1.69 × 10^9^ bases) (Supplementary Information 2).

#### Time-series analysis of microbial species composition

**Figure 2:**
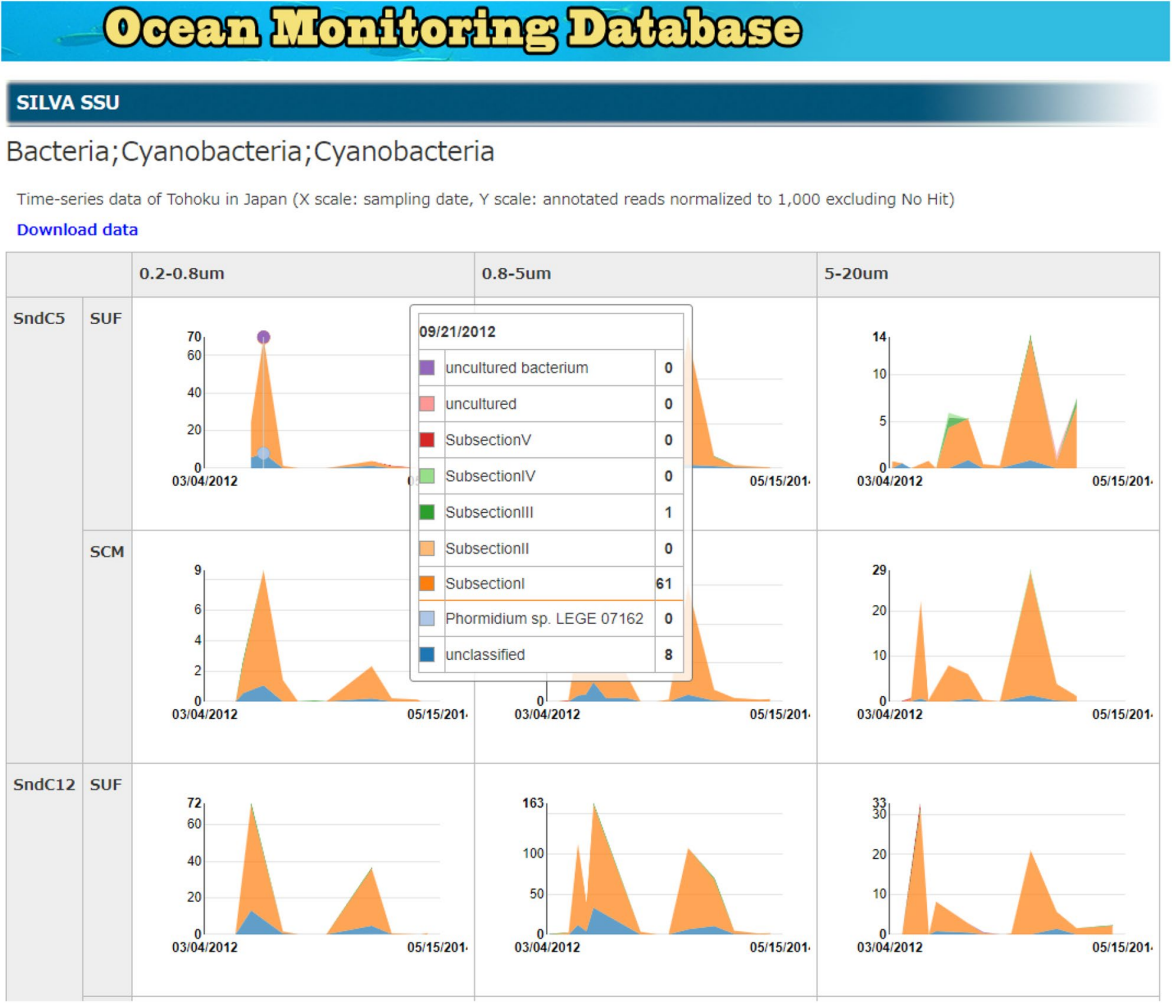
Time-series analysis of microbial communities along the Pacific coast of northeastern Japan. Each sampling point shows the number of ribosomal sequences normalized to 1000 (excluding no hits). Clicking on the graph at the website of Ocean Monitoring Database exhibits the next taxonomic levels. This figure shows an example of the change in *Cyanobacteria* communities over time from April 2012 to May 2014. SUF: surface layer (1 m), SCM: the subsurface chlorophyll-a maximum layer.

We performed microbial taxonomic assignments through analyses of amplicon and shotgun metagenomic sequence data using the SILVA and NCBI NT databases. For C5 and C12 samples from Sendai Bay and A4 and A21 samples from A-line, sufficient time-series data were available to plot changes in microbiota over time (Fig. 2). By clicking the displayed taxon at the website of Ocean Monitoring Database, the microbiota composition of lower taxa is revealed. For example, Fig. 2 shows that the cyanobacteria community increased in abundance during the summer.

**Figure 3:**
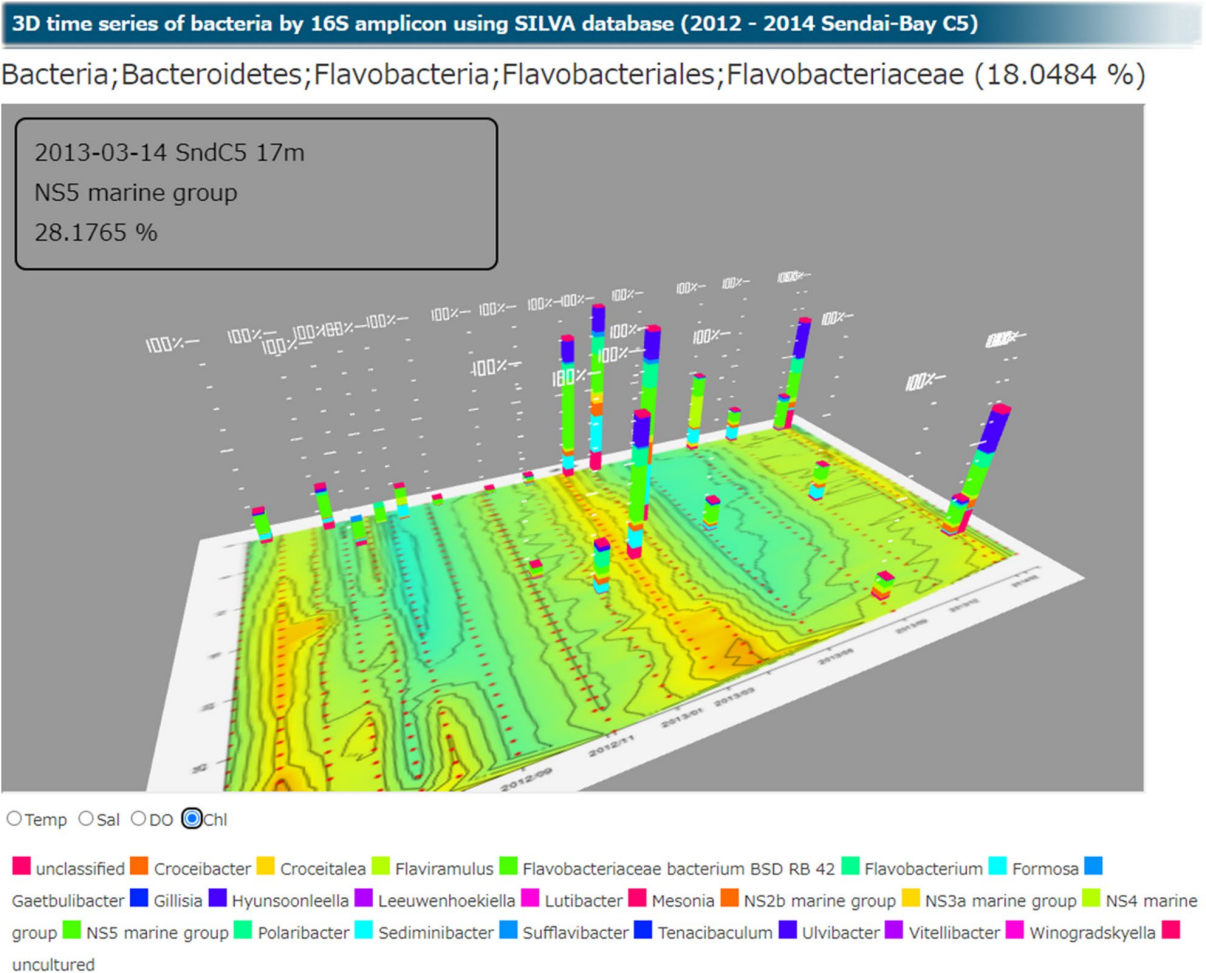
Three-dimensional (3D) display of microbial communities. 3D display of bacterial communities identified using 16S rRNA gene amplicon analysis of the Sendai Bay C5 samples from 2012 to 2014. The x-axis indicates the date, the y-axis indicates the water depth, and the z-axis indicates the percentage abundance of bacterial genera. The contour plot on the xy plane indicates the chlorophyll concentration. The composition of Flavobacteriaceae is shown as an example.

We acquired substantial 16S rRNA gene amplicon sequencing data at the C5 fixed point at Sendai Bay between 2012 and 2014. We generated a 3D graph to simultaneously display the date, water depth, and species composition at this site (Fig. 3). By clicking on the taxon shown in the graph at the website of Ocean Monitoring Database, the composition of the microbiota within a lower taxon is displayed. The 3D display is suitable for presenting a bird’s-eye view of the metagenomic data, which is extremely useful for visualizing and understanding the relationships among microbial communities among sampling points. This innovative function was incorporated into the metagenomic database. Figure 3 shows a contour map of chlorophyll concentrations on the x-axis and the proportion of microbial communities on the z-axis. The proportion of flavobacteria may increase following an increase in chlorophyll concentration during a spring bloom. For example, Buchan et al. reported that the proportion of flavobacteria increase late in a spring bloom^16^; our results show similar patterns (Fig. 3).

#### Digital DNA chip (DDC) database

**Figure 4:**
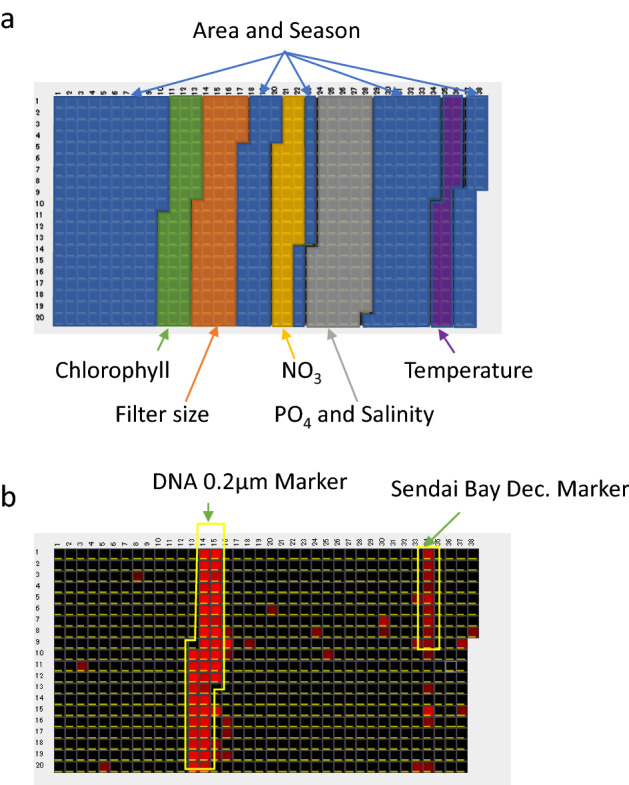
Visualization of metagenomics data using digital DNA chips. (**a**) Overview of in silico probes associated with the environmental factors on a digital DNA chip (See Supplementary Information 8 for details). (**b**) Digital DNA chip of shotgun metagenomics data of a 0.2–0.8-μm fraction of December 1, 2013, Sendai Bay C5. There are 748 probes, and spots that are positive for digital hybridization are shown in red. Negative spots are black. The hybridization positive probes are an indicator of environmental information of the sequence data.

A DDC analysis (DDCA) system is useful for visualizing the characteristics of shotgun metagenomic data as a microarray^17^ of, for example, filter size, water sampling point, water sampling time, temperature, salinity, and nitrate and phosphate concentrations (Fig. 4a). By mapping sequence data against the probe sets described above, which are associated with environmental factors, we predicted that sequence data would be more enriched and inclusive of environmental information. Figure 4b displays the DDCA shotgun metagenomic data of the 0.2–0.8-μm fraction of the C5 sample collected from Sendai Bay on December 1, 2013. The sample contains a bacterial-fraction DNA marker with filter sizes of 0.2–0.8 µm and a specific DNA marker for December in Sendai Bay. Even if there is only NGS data and no environmental information, just by looking at the digital DNA chip, we can assume this sample is extracted from 0.2 to 0.8 µm fraction and is from Sendai Bay (Fig. 4b).

#### Development of a shotgun metagenomic database

**Figure 5:**
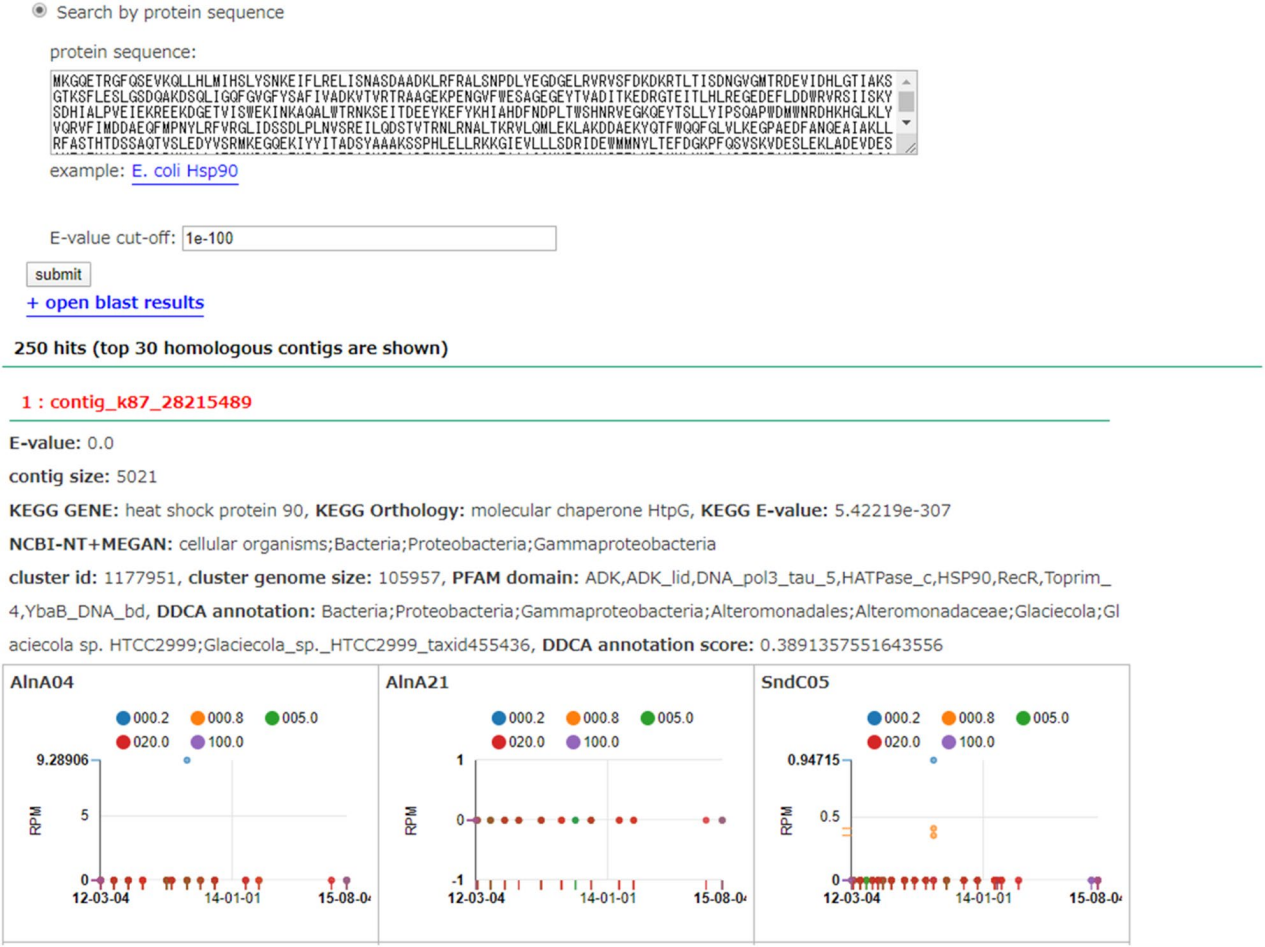
Search for homologous contigs to a query sequence and display of temporal variation patterns. Using nucleotide and amino acid sequences as queries, contigs homologous to the query sequence are identified using BLAST, and the temporal variation patterns and taxonomy information of the hit contigs are displayed.

We assembled the shotgun metagenomic sequence data using Megahit version 1.0.2^18^. There were 57.95 M contigs, with an N50 of 995 bp, a maximum length of 307,212 bp, and a total of 12.39 Gbp (Supplementary Information 3). We calculated the abundance pattern of each contig. Those contigs whose appearance pattern matched with a Pearson correlation coefficient of ≥ 0.95 were clustered into a MAG. We next added the annotation of assembled contigs to the results of the BLAST search of the NCBI NT database and using classification by clustering with Pfam (CCP). This novel annotation method is described below. We developed the database showing the abundance pattern of homologous contigs against a queried sequence by BLAST for each sampling point and filter size (Fig. 5). For example, we found novel *PolD* families using this database.

#### Development of a new annotation method for metagenome contigs

**Figure 6:**
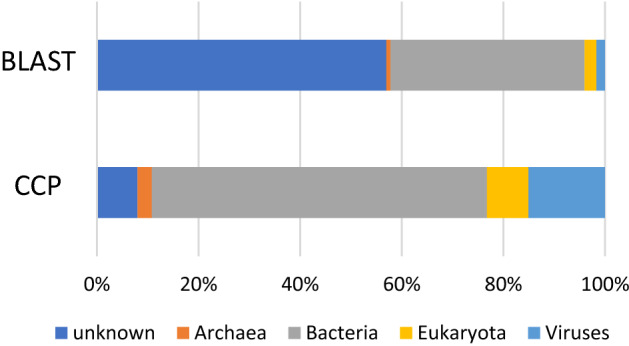
Comparison between BLAST and CCP annotation results at the super-kingdom level. Comparison of classification results using BLAST to annotate contigs and classification by clustering with Pfam (CCP); the percentage of unknowns was 57% for BLAST and 8% for CCP.

We annotated the assembled contigs using BLAST to analyze the NCBI NT database. However, we were unable to annotate > 50% of the contigs (Fig. 6). Therefore, we developed a novel method, i.e., CCP, to annotate contigs according to their species names. Analysis using a single contig generally does not provide sufficient information for assigning an annotation. However, CCP assigns the appropriate annotation to the sequence because it aggregates the Pfam information of all contigs in a MAG. CCP annotates a MAG by comparing the similarity to the reference genome. Comparing the nucleotide sequences of a MAG directly using blastn to analyze the NCBI NT database shows relatively low homology to de novo virus sequences.

**Figure 7:**
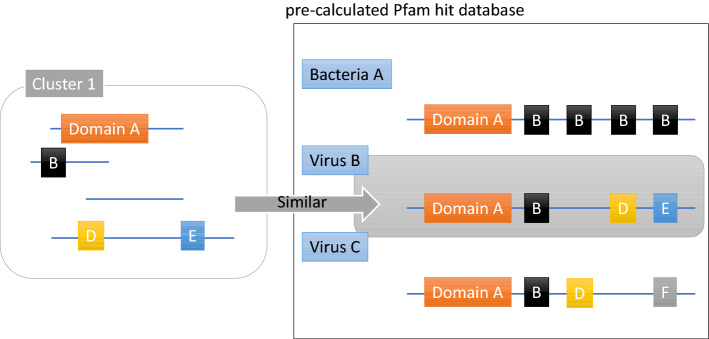
Overview of CCP. Flowchart of the search of the Pfam domain against known genomes of viruses, bacteria, archaea, and eukaryotes included in RefSeq to create a Pfam hit database. The Pfam domains were searched in metagenome-assembled genome (MAG) units and the known genomes whose type and number of Pfam domains are closest to the MAG.

However, a Pfam domain search using HMMER, which employs a different principle^19^ than BLAST, often detects more informative sequences, even those of viruses. For example, phylogenetic trees constructed according to the type and number of Pfam domains of individual bacterial genomes and those of higher eukaryotes such as humans closely approximate those generated using existing phylogenetic trees^20^. Thus, genomes with a similar Pfam domain may represent phylogenetically closely related species. We, therefore, searched the Pfam domains for reference genomes of viruses, bacteria, archaea, and eukaryotes included in RefSeq (as of August 31, 2015). We next calculated the number of domains for each species and constructed a CCP database. The types and numbers of Pfam domains contained in the contig obtained from the metagenome were summarized in MAG units. We compared the results using the CCP database and annotated the known genomes with the closest correlation coefficient (Fig. 7).

By annotating the top 10,000 contigs with the highest abundance in our database using CCP, > 90% of the contigs were explained (Fig. 6). In contrast, the BLAST species search (the existing method) returned < 50% annotated contigs (Fig. 6), indicating that CCP classified the contigs more comprehensively. In particular, virus annotation significantly improved from 2 to > 15%, indicating that CCP is a robust method, particularly when applied to the identification of virus annotation. We next compared the agreement between CCP and BLAST annotations using contigs annotated using both CCP and BLAST (Supplementary Information 4). The virus-level agreement between CCP and BLAST was 89.6%, and the kingdom-level agreement was 76.9%. It was difficult to determine whether the contig represented a virus using BLAST; however, CCP showed higher accuracy.

### Shotgun metagenomic analysis

#### Periodicity of metagenomic data

**Figure 8:**
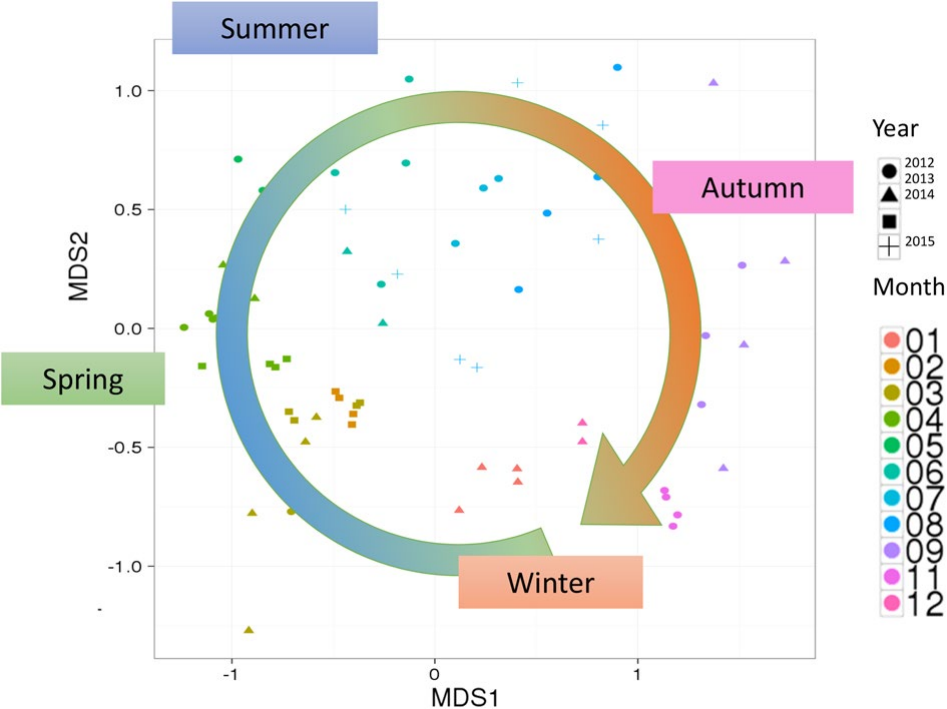
Multidimensional scaling (MDS) plot as a function of the abundance of contigs**.** MDS plots of bacterial fractions (0.2–0.8 µm) of shotgun metagenomic data from 2012 to 2015 acquired from Sendai Bay according to the pattern of abundance of assembled contigs.

**Figure 9:**
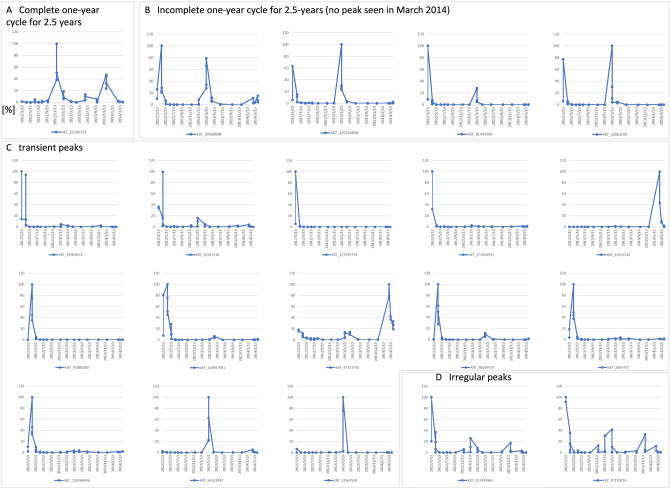
Variation patterns of contigs in the top 20 most abundant metagenome-assembled genomes (MAGs).The top 20 MAGs in the bacterial fractions of Sendai Bay C5 and C12 from March 13, 2012, to April 2, 2014, were classified as follows: (**a**) complete 1-year cycle for 2.5 years, (**b**) Incomplete 1-year cycle for 2.5 years, (**c**) transient peaks, and (**d**) irregular peaks. A peak within 1 month of ≥ 25% relative to the previous year’s peak was considered cyclical.

For the bacterial fraction (0.2–0.8 µm) of the shotgun metagenomic data from Sendai Bay, we generated a multidimensional scaling (MDS) plot according to the pattern of the abundance of the assembled contigs (Fig. 8). The MDS plot shows similarities among the samples collected during the same month during different years. Furthermore, the plot reveals that the shotgun metagenomic data exhibit an annual seasonal cycle like the 18S rRNA amplicon data^10^. However, we did not observe the same annual cycle among all contigs. We, therefore, extracted contigs included in the top 20 highly abundant MAGs from the bacterial fractions of Sendai Bay samples collected from March 2012 to April 2014 and plotted the fluctuation patterns. Only one such contig showed a complete 2-year cycle (Fig. 9a), and four contigs showed an incomplete 2-year cycle with peaks in March 2012 and 2013 but not in 2014 (Fig. 9b). Furthermore, 13 MAGs showed a transient pattern (Fig. 9c), and two MAGs showed peaks with irregular patterns (Fig. 9d). These results suggest that marine microbial communities generally undergo an annual cycle.

#### Identification of repeat sequences in the metagenomes

During the collection and analysis of the DNA sequencing data, we identified a number of repeat sequences in the metagenomes as follows: (TAG)n, (TGA)n, (GAA)n, and (ACA)n microsatellites. We then determined the frequencies and highest numbers of (TAG)n repeats as a function of filter size. We found that the (TAG)n repeats included up to 7.5% of the 5–20-μm fraction (Supplementary Information 5a,b). To investigate whether this was a characteristic feature of the northeastern coastal region of Japan, we analyzed the shotgun metagenomic sequence data of Tara Oceans^21,22^. As shown in Supplementary Information 5c,d, Tara Oceans data contained up to 1.9% of TAG repeats.

**Figure 10:**
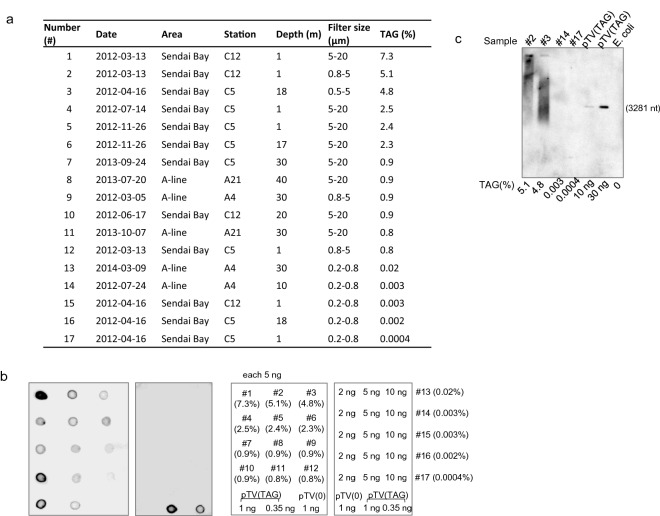
Detection of TAG repeats using Southern blot and dot-blot hybridization analyses. (**a**) Contents of the TAG repeats of the samples according to next-generation sequencing analysis. (**b**) Dot-blot analyses. The sample numbers and their amounts, (right side) correspond to the signals of each dot in the left panels. The intact pTV119N plasmid without an insert indicates pTV(0). The calculated contents of TAG repeats (%) are indicated in parentheses. (**c**) Southern blot analysis of EcoRI-digested samples subjected to 0.8% agarose gel electrophoresis. The plasmid pTV (TAG) (0.35 ng and 1 ng) served as a positive control. *E. coli* genomic DNA served as a negative control. The calculated contents of TAG repeats (%) are indicated on the bottom of each graph. The length (nt) of the TAG-repeated fragment excised from pTV (TAG) is shown on the right.

To determine whether these (TAG)n repeats represented artifacts of the NGS method, we performed Southern blot and dot-blot hybridization analyses of the DNA samples extracted from seawater (Fig. 10). The dot-blot hybridization experiment analyzed 13 different samples with various content rates (Fig. 10a). We detected signals from the eight samples containing the (TAG)n that were repeated in > 0.9% of the labeled d54-mer with the (TAG)_18_ repeat. In contrast, six samples with a low content (> 0.2%) were negative (Fig. 10b). To determine whether these repeated sequences originated from a single locus or multiple loci, we performed Southern blot analysis (Fig. 10c, Supplementary Information 6) using two samples with high contents of (TAG)n repeats. A (TAG)n representing a single locus is detectable as a discrete band versus the diffuse bands exhibited by two samples with a high content of (TAG)n repeats. The data (Fig. 10c) suggest that the (TAG)n repeats were derived from multiple loci of distinct genomes. Samples with low numbers of (TAG)n repeats were negative.

These results reveal for the first time that such repeat sequences are abundant in the genomes of marine microorganisms. However, their species of origin and functional roles were not identified here. The repeat sequences found in *Escherichia coli*^23^, subsequently called CRISPR, led to fundamental discoveries that are essential in the field of genetic engineering^24^. Thus, understanding the biological significance of trinucleotide repeats in marine microorganisms is of particular importance and may reveal a new research frontier.

## Conclusion

In this study, we present marine shotgun metagenomic time-series data acquired using a novel annotation method. We used these data to construct a comprehensive database that includes a search function for BLAST analysis and can be browsed in a 3D view. This unique database, which includes marine metagenomic data focusing on a specific area, will serve as a valuable tool. For example, we identified the seasonal periodicity of metagenome profiles. In a future study, we expect to develop a method to predict microorganism behaviors. Furthermore, we used shotgun metagenomic sequencing to identify (TAG)n repeats characteristic of the genomes of marine microorganisms; these repeats could not be identified using amplicon sequencing. Such repeats may contribute to the higher-order genomic structures of marine microorganisms. We expect that our present findings will lead to significant discoveries associated with the dynamics of microbial communities.

## Methods

### Sample collection and DNA extraction

Between March 2012 and May 2016, seawater samples were collected from locations around Sendai Bay, Ofunato Bay, and A-line (Fig. 1). We conducted sampling investigations at Sendai Bay once a month or more from 2012 to 2015 and collected seawater samples at two sites (C5: 38°00′N, 141°00′E, C12: 38°00′N, 141°30′E) at the surface and SCM layers. We conducted sampling along the A-line (offshore area) once every 3 months from 2012 to 2015 and collected seawater samples at two sites (A4: 42°15′N, 145°15′E, A21: 38°00′N, 147°15′E) at the surface and SCM layers. In 2016, surface water samples were collected once every 2 weeks at a single site at Ofunato Bay during the spring bloom season (Supplementary Information 7). Water samples were collected using a CTDO (SeaBird SBE911 + SBE43) rosette system equipped with 10-L X-Niskin bottles or a 6-L Van Dorn water sampler.

Water temperature, salinity, chlorophyll-a concentration, and dissolved oxygen concentration were measured at the time of sampling onsite using an Aqua Quality Sensor (AAQ-1186; JFE Advantech, Japan). The chlorophyll-a concentration was calibrated using a fluorescence method^13^, and the dissolved oxygen concentration was calibrated using the Winkler method^13^. Water samples (approximately 9 L) were cooled and placed in the dark immediately following collection. Large organisms such as zooplankton were removed from the samples using a 100-μm plankton net. The samples containing materials retained on the plankton net were designated as the 100-μm fraction. Fractions (0.2–0.8, 0.8–5, 5–20, and 20–100 μm) were sequentially passed through a 20-μm pore nylon net filter (47-mm diameter) and 5-μm, 0.8-μm, and 0.2-μm pore nucleopore filters (142-mm diameter) (GS, Millipore) using a peristaltic pump^25^. For shotgun and 16S rRNA gene amplicon sequencing of the 0.2-μm fraction, DNA was extracted from the filters and the retained materials using a PowerWater DNA Isolation Kit (Mo Bio). For the > 0.2-μm fraction used for 16S rRNA gene amplicon analysis, 300–500 mL of sampled water was passed through a membrane filter (0.22–μm pore; GS, Millipore) and DNA was extracted using a FAST DNA-SPIN kit for soil (MP. Biomedicals) following the manufacturer’s instructions to avoid the inhibitory effect of high concentrations of suspended matter in the bottom-layer water. For the < 5-μm fraction used for 18S rRNA gene amplicon analysis, seawater was first passed through a 5-µm-mesh plankton net, and pico- and nanophytoplankton cells in the filtrate were concentrated using tangential flow filtration equipped with a regenerated cellulose membrane (100-kD MWCO). DNA was extracted using an alkaline lysis method^27^.

### Multitag Illumina sequencing of 16S rRNA genes

PCR was performed using 27Fmod and 338R primers^26^ modified by adding 33- and 34-bp nucleotides, respectively. Molecular identifier (MID) tags were subsequently added. The PCR process consisted of initial denaturation at 95 °C for 2 min, followed by 20 cycles of denaturation at 95 °C for 30 s, annealing at 52 °C for 30 s, and extension at 72 °C for 30 s. A final elongation step was performed for 1 min at 72 °C. The product was visualized on an agarose gel using SYBR Safe (Life Technologies), excised, purified, and quantified using a QuantiFluor (Promega). Approximately 10 ng of the first PCR product was used as a template for the second PCR, to which MID tags were added using a forward (59 bp) and reverse (54 bp) primer set with an initial denaturation at 98 °C for 30 s, followed by 12 cycles of denaturation at 98 °C for 10 s, annealing at 60 °C for 30 s, and extension at 72 °C for 30 s. A final elongation step was performed for 1 min at 72 °C. The PCR programs were determined following the manufacturer’s instructions (FASMAC Co. Ltd). Sequencing was performed using an Illumina MiSeq apparatus(300-bp paired-end run) at FASMAC Co. Ltd. Average sequenced reads and bases are shown in Supplementary Information 2.

### Multitag pyrosequencing analysis of 18S rRNA genes

DNA was amplified using GenomiPhi version 2 to produce sufficient amounts of DNA for the downstream procedure. Eukaryotic 18S rDNA was amplified using 545F and 1119R primers^28^ ligated to MID tags. The amplicons (approximately 580 bp in size) were electrophoretically separated using agarose gel, purified using a QIAquick Gel Extraction Kit (QIAGEN), and analyzed using a GS Jr. 454-pyrosequencer at the University of Tsukuba. Average sequenced reads and bases are shown in Supplementary Information 2.

### Library preparation for shotgun metagenomic analysis

The library was prepared using a Nextera XT DNA Library Preparation Kit (Illumina) according to the manufacturer’s protocol. After determining the library concentration at the tape station, 100-bp paired-end shotgun sequencing data were obtained using an Illumina HiSeq 2000 at the University of Tokyo. Average sequenced reads and bases are shown in Supplementary Information 2.

### Species composition analysis of 16S and 18S rRNA gene sequences using amplicon sequencing and shotgun sequencing

A homology search of the SILVA database (SSU_Ref_NR version 119)^29^or NCBI NT database (as of December 11, 2015)^30^ was performed using blastn. Blastn hits with bit scores of > 100 were extracted, and microbial taxonomic assignment was performed using the LCA method with metagenome ~ silva_SSU + LSU script of Portable Pipeline (https://github.com/c2997108/OpenPortablePipeline).

### DDCA

Shotgun metagenomic data for the microarray-like image was visualized using marine feature chips from DDCA (Japan Software Management Co., Ltd.).

### Shotgun data analysis pipeline

#### Assembly and MAG creation

The shotgun metagenomic data were assembled using Megahit version 1.0.2^18^ to create contigs, which were mapped using BWA mem version 0.7.10^31^ to determine the number of hits for each contig (using the default option). Mapped reads with > 90% homology to the assembled contigs were extracted, and the number of reads that hit each contig was counted. The copy number per 1 L of seawater was calculated using an equation incorporating the amount of DNA, the total number of reads, and the number of reads that hit each contig as follows:

$$Copy \; number \;per \;1 \;L =Total \;DNA\; weight \times \frac{number\; of \;mapped \;reads}{number\; of \;total \;reads}\times \frac{6.02 \times {10}^{23}}{616 \times MAG \;length \left(bp\right)} \times \frac{1}{water \;volume\; (L)}$$

The top 5 million contigs were extracted with the highest number of hits to reduce computational time. These contigs were used for subsequent analysis. The MAG was created using the method described by Nielsen et al.^5^ by clustering contigs with similar appearance patterns and a Pearson correlation coefficient of ≥ 0.95.

#### CCP species classification

The Pfam domain was searched for known genomes of 4,534 viruses, 29,769 bacteria, 444 archaea, and 12,940 eukaryotes included in RefSeq (ftp://ftp.ncbi.nih.gov/genomes/refseq/). To search the Pfam domain, the number of domains held for each strain was calculated using pfam_scan version 1.6^32^. Then, the Pfam domains in the contig were searched in MAG units, the types and numbers of Pfam domains of each MAG were identified and compared with the known genome, and the known genome with the closest correlation coefficient was annotated (Fig. 6). The series of analysis pipelines is available at https://github.com/jsm-nbb/ccp.

#### BLAST annotation

The NCBI NT database was searched using blastn (BLAST + version 2.3.0^33^) for each contig with -outfmt 6 and -num_threads 8 options. The results of the BLAST hits were tabulated using the LCA method of MEGAN6 with default options^34^. For BLAST–MAG annotation, the annotation of the longest contig in the MAG was adopted.

### Search for repeat sequences

The percentage of repeat sequences in the reads was calculated. We defined reads that contained repeats of > 70 bp of 100 bp as repeat sequences. All repeats in 2–10 base units were calculated, and the most common repeat was found to be (TAG)n (stop codon depending on the reading frame), followed by TGA (stop codon depending on the reading frame), GAA, and ACA. The same was applied to the shotgun sequence data of the Tara Oceans project (Accession: PRJEB1787, PRJEB1788, and PRJEB4352)^21,22^ to identify the most frequent TAG repeats.

### Southern blot and dot-blot hybridizations

Restriction endonucleases were purchased from New England Biolabs. The synthetic oligonucleotide (5′-dCGCGAAGCTTAGTAGTAGTAGTAGTAGTAGTAGTAGTAGTAGTAGTAGTAGTAGTAGTAGTAGAAGCTTGGG-3′ (repeated TAG in bold, *Bam*HI recognition site underlined) and its complementary oligonucleotide were annealed in 40 mM Tris–acetate (pH 7.8) and 0.5 mM magnesium acetate. The annealed fragment was digested using *Bam*HI and ligated to the corresponding site of pTV119N (Takara Bio). The plasmid harboring two tandemly ligated fragments, including 36 TAG repeats (3.3%), was selected as a positive control designated pTV(TAG). The intact pTV119N plasmid and genomic DNA of *E. coli* JM109 served as negative controls. DNA (300 ng) was digested using *Eco*RI, and the fragments were separated using 0.8% agarose gel electrophoresis and transferred to a nylon membrane (Biodyne PLUS, Pall corporation). Five nanomoles of 5′-biotinylated probes (dCTACTACTACTACTACTACTACTACTACTACTACTACTACTACTACTACTACTA) were hybridized at 50 °C for 16 h with DNA fragments on the nylon membrane, which was washed twice at 25 °C for 15 min. A chemiluminescent nucleic acid detection module kit (Thermo Fisher Scientific) and LAS-3000 (GE Healthcare) were used for signal detection according to the manufacturer’s instructions. Dot-blot hybridization was performed to examine samples containing smaller amounts. DNA was diluted with 0.4 M NaOH, and aliquots (5 μl) were spotted on the nylon membrane. After neutralization, hybridization was performed at 55 °C for 16 h, and the nylon membrane was washed twice at 55 °C for 15 min. The detection step is described above.

## Supplementary Information

Supplementary Information 1.

Supplementary Information 2.

## Data Availability

CCP is available for download at https://github.com/jsm-nbb/ccp. All sequencing data are available from Ocean Monitoring Database and are registered in the SRA under the Accession Number DRA005425.
