# Supplementary material for: Development of a time-series shotgun metagenomics database for monitoring microbial communities at the Pacific coast of Japan
Source: Sci Rep. 2021 Jun 9;11:12222. doi: 10.1038/s41598-021-91615-3 (PMC8190148; doi:10.1038/s41598-021-91615-3)
Supplement: Supplementary file 2 — Supplementary Information 2. [file 41598_2021_91615_MOESM2_ESM.pptx]

## Slide 1
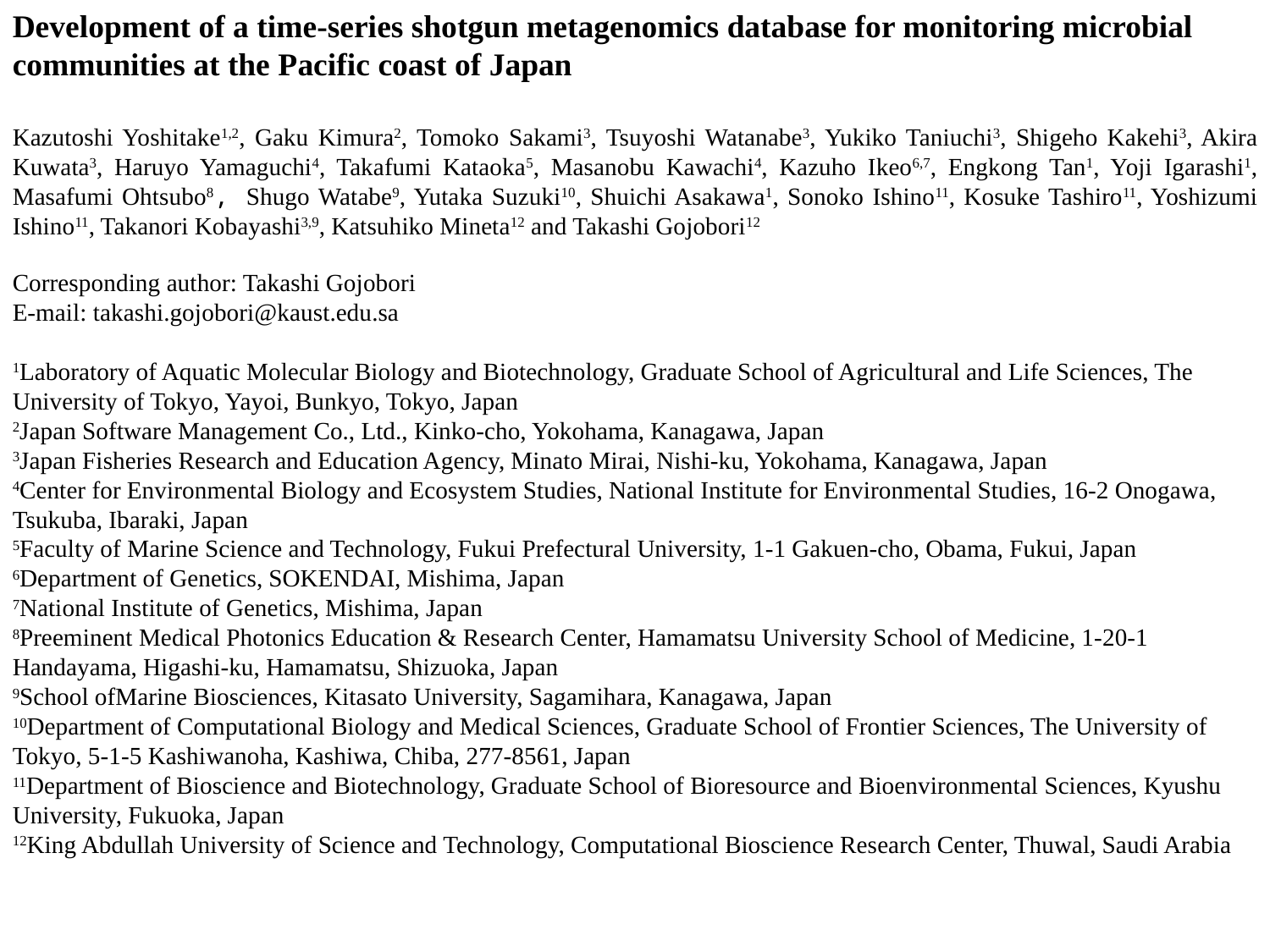

Development of a time-series shotgun metagenomics database for monitoring microbial communities at the Pacific coast of Japan
Kazutoshi Yoshitake1,2, Gaku Kimura2, Tomoko Sakami3, Tsuyoshi Watanabe3, Yukiko Taniuchi3, Shigeho Kakehi3, Akira Kuwata3, Haruyo Yamaguchi4, Takafumi Kataoka5, Masanobu Kawachi4, Kazuho Ikeo6,7, Engkong Tan1, Yoji Igarashi1, Masafumi Ohtsubo8, Shugo Watabe9, Yutaka Suzuki10, Shuichi Asakawa1, Sonoko Ishino11, Kosuke Tashiro11, Yoshizumi Ishino11, Takanori Kobayashi3,9, Katsuhiko Mineta12 and Takashi Gojobori12
Corresponding author: Takashi Gojobori
E-mail: takashi.gojobori@kaust.edu.sa
1Laboratory of Aquatic Molecular Biology and Biotechnology, Graduate School of Agricultural and Life Sciences, The University of Tokyo, Yayoi, Bunkyo, Tokyo, Japan
2Japan Software Management Co., Ltd., Kinko-cho, Yokohama, Kanagawa, Japan
3Japan Fisheries Research and Education Agency, Minato Mirai, Nishi-ku, Yokohama, Kanagawa, Japan
4Center for Environmental Biology and Ecosystem Studies, National Institute for Environmental Studies, 16-2 Onogawa, Tsukuba, Ibaraki, Japan
5Faculty of Marine Science and Technology, Fukui Prefectural University, 1-1 Gakuen-cho, Obama, Fukui, Japan
6Department of Genetics, SOKENDAI, Mishima, Japan
7National Institute of Genetics, Mishima, Japan
8Preeminent Medical Photonics Education & Research Center, Hamamatsu University School of Medicine, 1-20-1 Handayama, Higashi-ku, Hamamatsu, Shizuoka, Japan
9School ofMarine Biosciences, Kitasato University, Sagamihara, Kanagawa, Japan
10Department of Computational Biology and Medical Sciences, Graduate School of Frontier Sciences, The University of Tokyo, 5-1-5 Kashiwanoha, Kashiwa, Chiba, 277-8561, Japan
11Department of Bioscience and Biotechnology, Graduate School of Bioresource and Bioenvironmental Sciences, Kyushu University, Fukuoka, Japan
12King Abdullah University of Science and Technology, Computational Bioscience Research Center, Thuwal, Saudi Arabia

## Slide 2
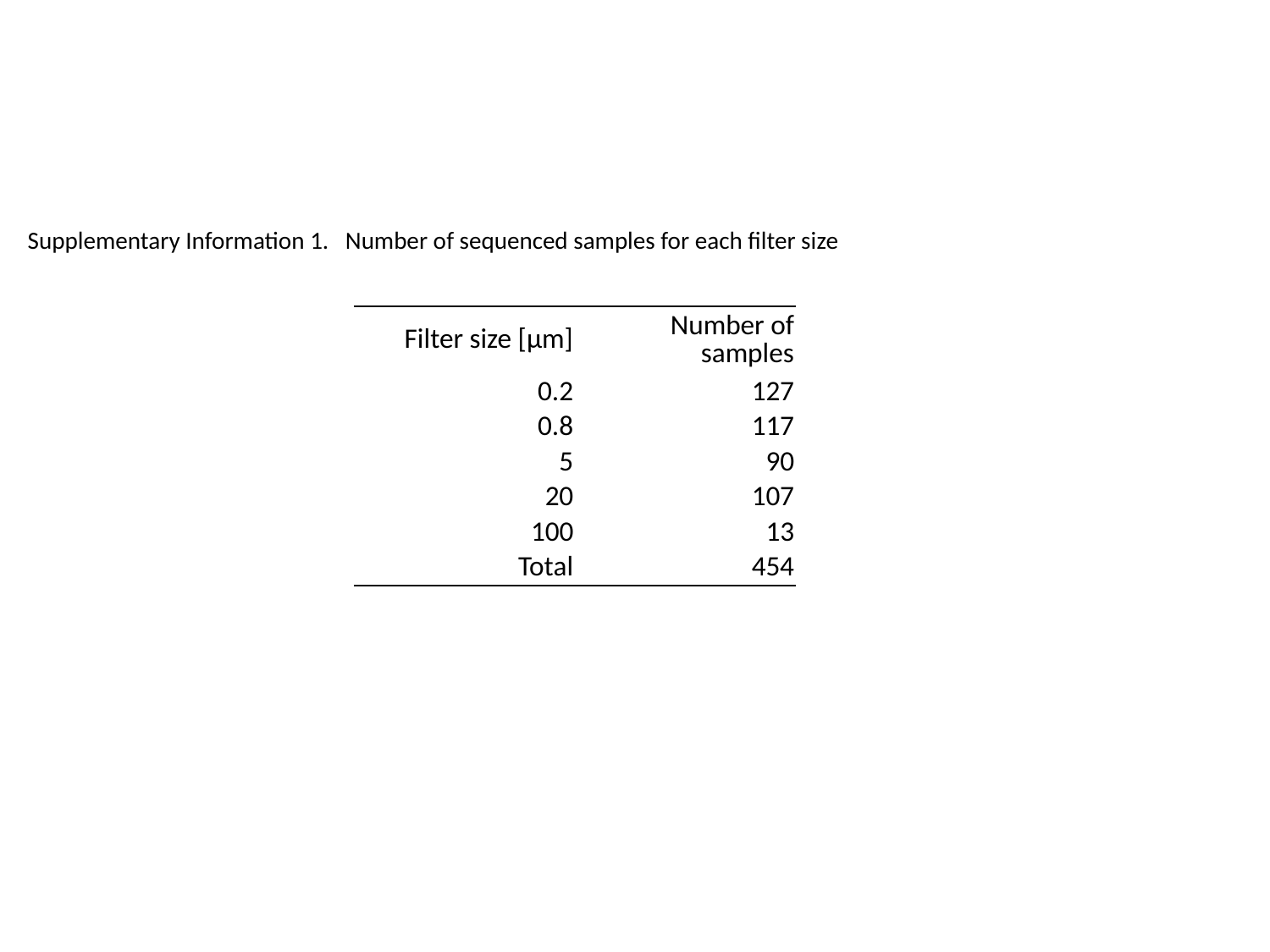

Supplementary Information 1. Number of sequenced samples for each filter size
| Filter size [µm] | Number of samples |
| --- | --- |
| 0.2 | 127 |
| 0.8 | 117 |
| 5 | 90 |
| 20 | 107 |
| 100 | 13 |
| Total | 454 |

## Slide 3
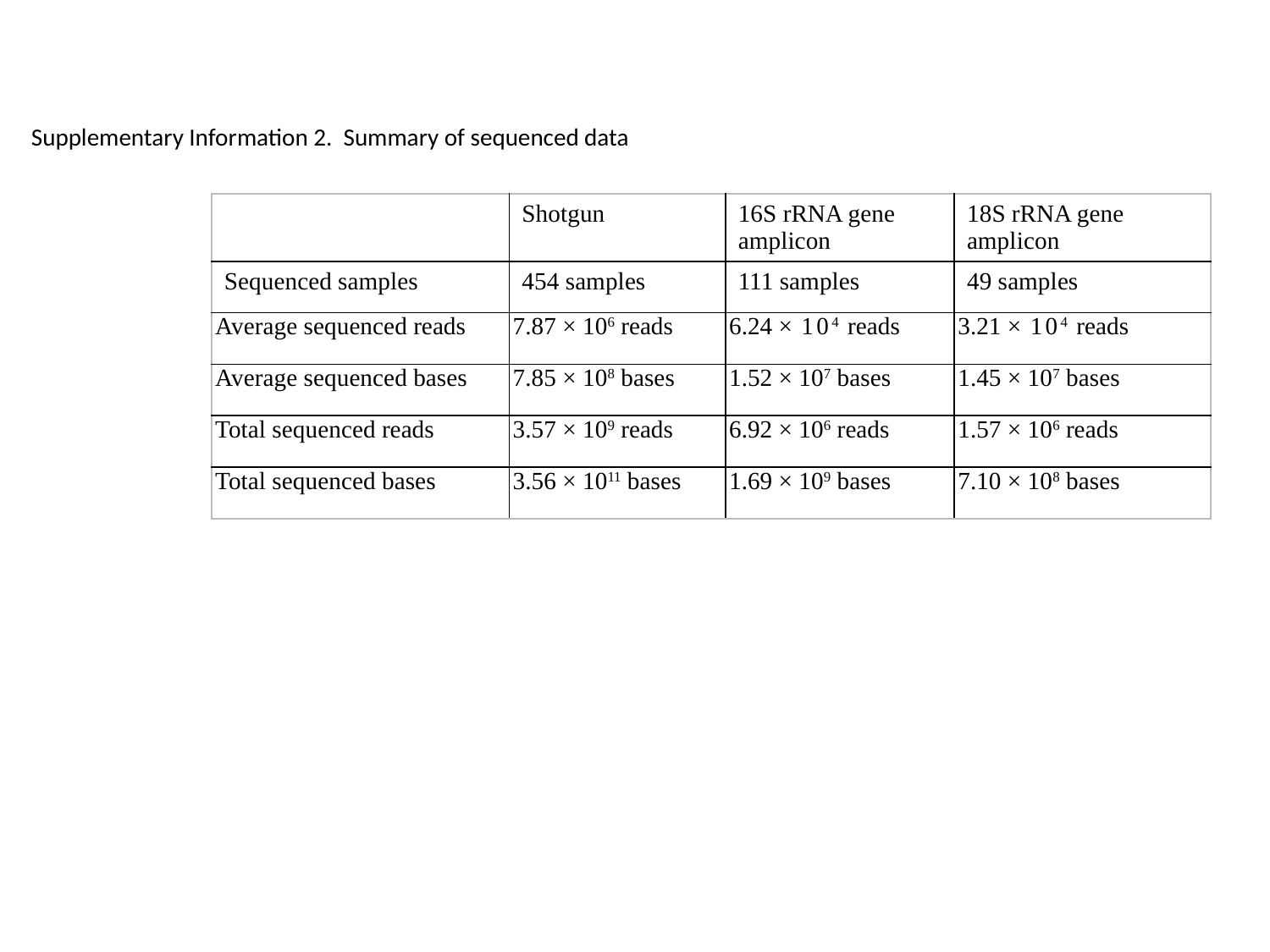

Supplementary Information 2. Summary of sequenced data
| | Shotgun | 16S rRNA gene amplicon | 18S rRNA gene amplicon |
| --- | --- | --- | --- |
| Sequenced samples | 454 samples | 111 samples | 49 samples |
| Average sequenced reads | 7.87 × 10^6^ reads | 6.24 × 10^4^ reads | 3.21 × 10^4^ reads |
| Average sequenced bases | 7.85 × 108 bases | 1.52 × 107 bases | 1.45 × 107 bases |
| Total sequenced reads | 3.57 × 10^9^ reads | 6.92 × 10^6^ reads | 1.57 × 10^6^ reads |
| Total sequenced bases | 3.56 × 1011 bases | 1.69 × 109 bases | 7.10 × 108 bases |

## Slide 4
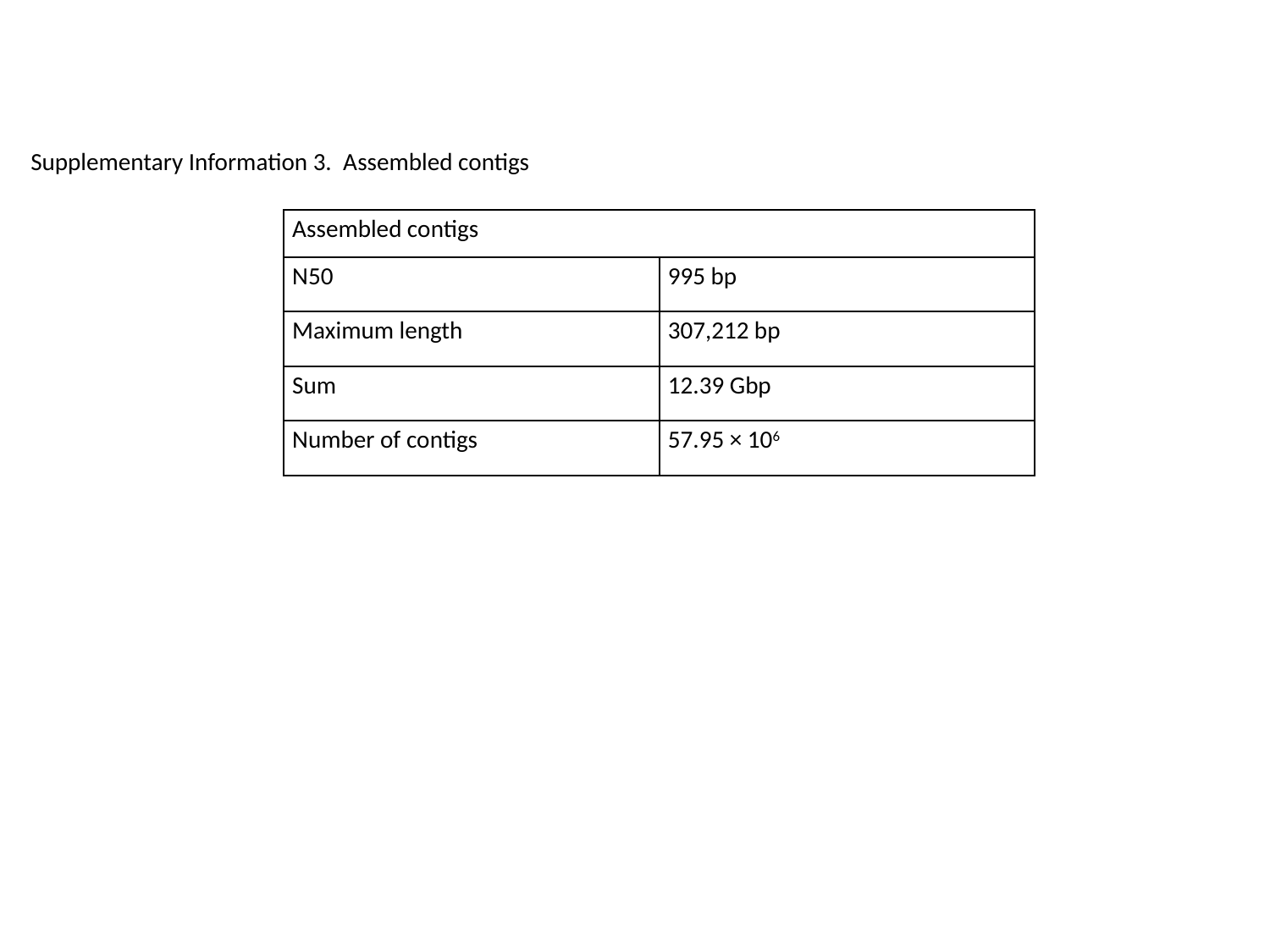

Supplementary Information 3. Assembled contigs
| Assembled contigs | |
| --- | --- |
| N50 | 995 bp |
| Maximum length | 307,212 bp |
| Sum | 12.39 Gbp |
| Number of contigs | 57.95 × 106 |

## Slide 5
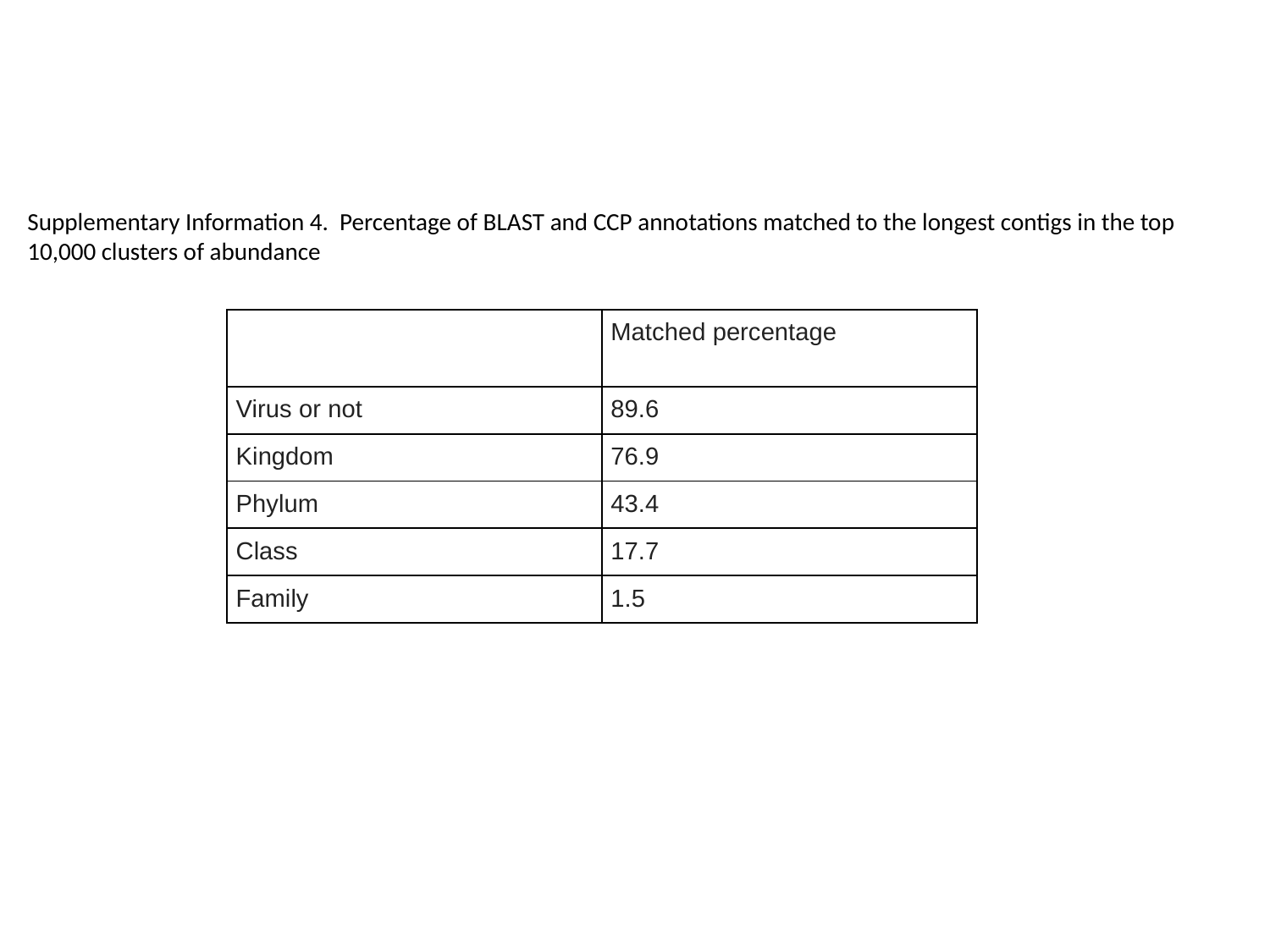

Supplementary Information 4. Percentage of BLAST and CCP annotations matched to the longest contigs in the top 10,000 clusters of abundance
| | Matched percentage |
| --- | --- |
| Virus or not | 89.6 |
| Kingdom | 76.9 |
| Phylum | 43.4 |
| Class | 17.7 |
| Family | 1.5 |

## Slide 6
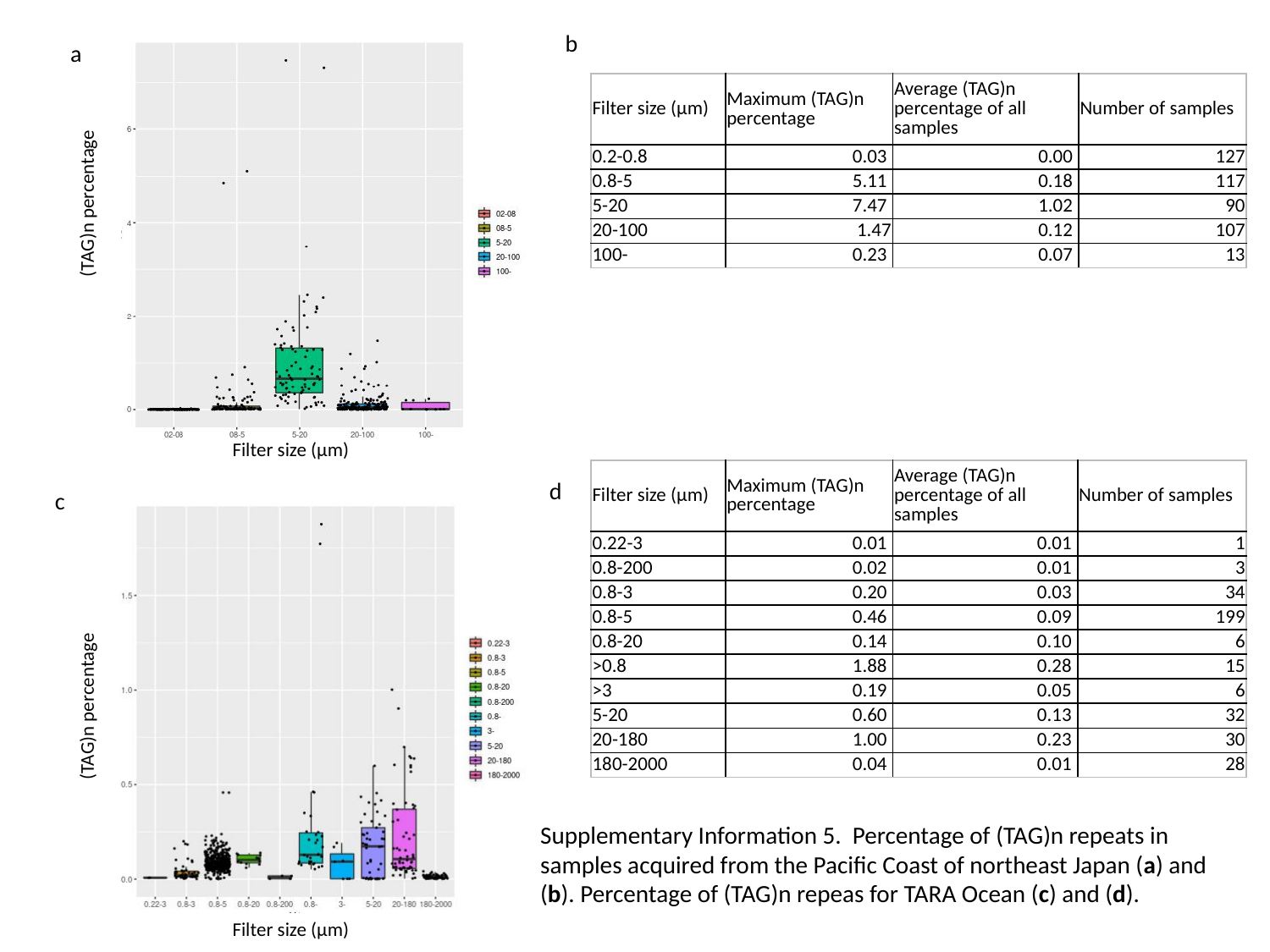

b
a
| Filter size (µm) | Maximum (TAG)n percentage | Average (TAG)n percentage of all samples | Number of samples |
| --- | --- | --- | --- |
| 0.2-0.8 | 0.03 | 0.00 | 127 |
| 0.8-5 | 5.11 | 0.18 | 117 |
| 5-20 | 7.47 | 1.02 | 90 |
| 20-100 | 1.47 | 0.12 | 107 |
| 100- | 0.23 | 0.07 | 13 |
(TAG)n percentage
Filter size (µm)
| Filter size (µm) | Maximum (TAG)n percentage | Average (TAG)n percentage of all samples | Number of samples |
| --- | --- | --- | --- |
| 0.22-3 | 0.01 | 0.01 | 1 |
| 0.8-200 | 0.02 | 0.01 | 3 |
| 0.8-3 | 0.20 | 0.03 | 34 |
| 0.8-5 | 0.46 | 0.09 | 199 |
| 0.8-20 | 0.14 | 0.10 | 6 |
| >0.8 | 1.88 | 0.28 | 15 |
| >3 | 0.19 | 0.05 | 6 |
| 5-20 | 0.60 | 0.13 | 32 |
| 20-180 | 1.00 | 0.23 | 30 |
| 180-2000 | 0.04 | 0.01 | 28 |
d
c
(TAG)n percentage
Supplementary Information 5. Percentage of (TAG)n repeats in samples acquired from the Pacific Coast of northeast Japan (a) and (b). Percentage of (TAG)n repeas for TARA Ocean (c) and (d).
Filter size (µm)

## Slide 7
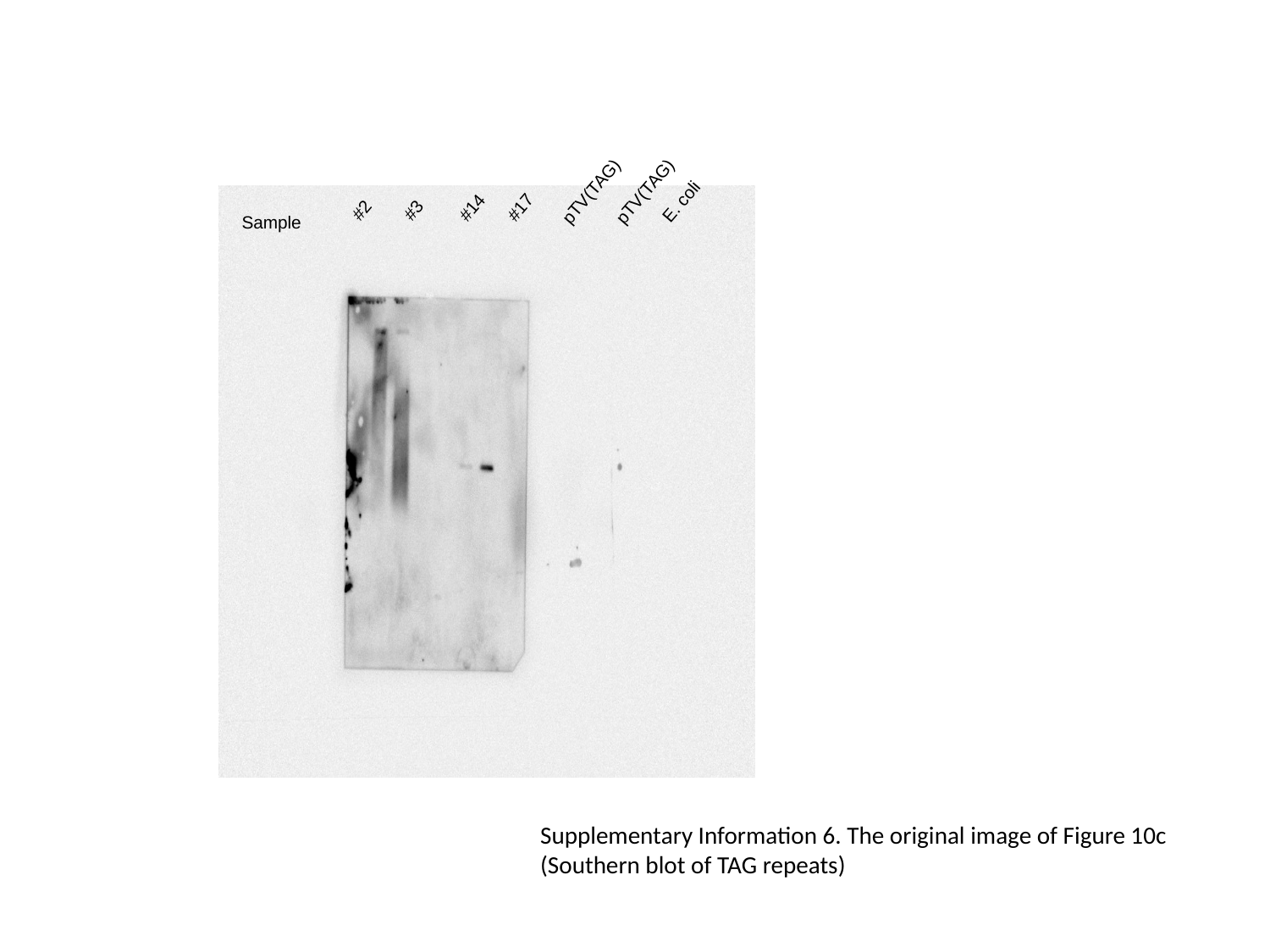

pTV(TAG)
pTV(TAG)
E. coli
#14
#17
#2
#3
Sample
Filter size (µm)
d
Supplementary Information 6. The original image of Figure 10c (Southern blot of TAG repeats)

## Slide 8
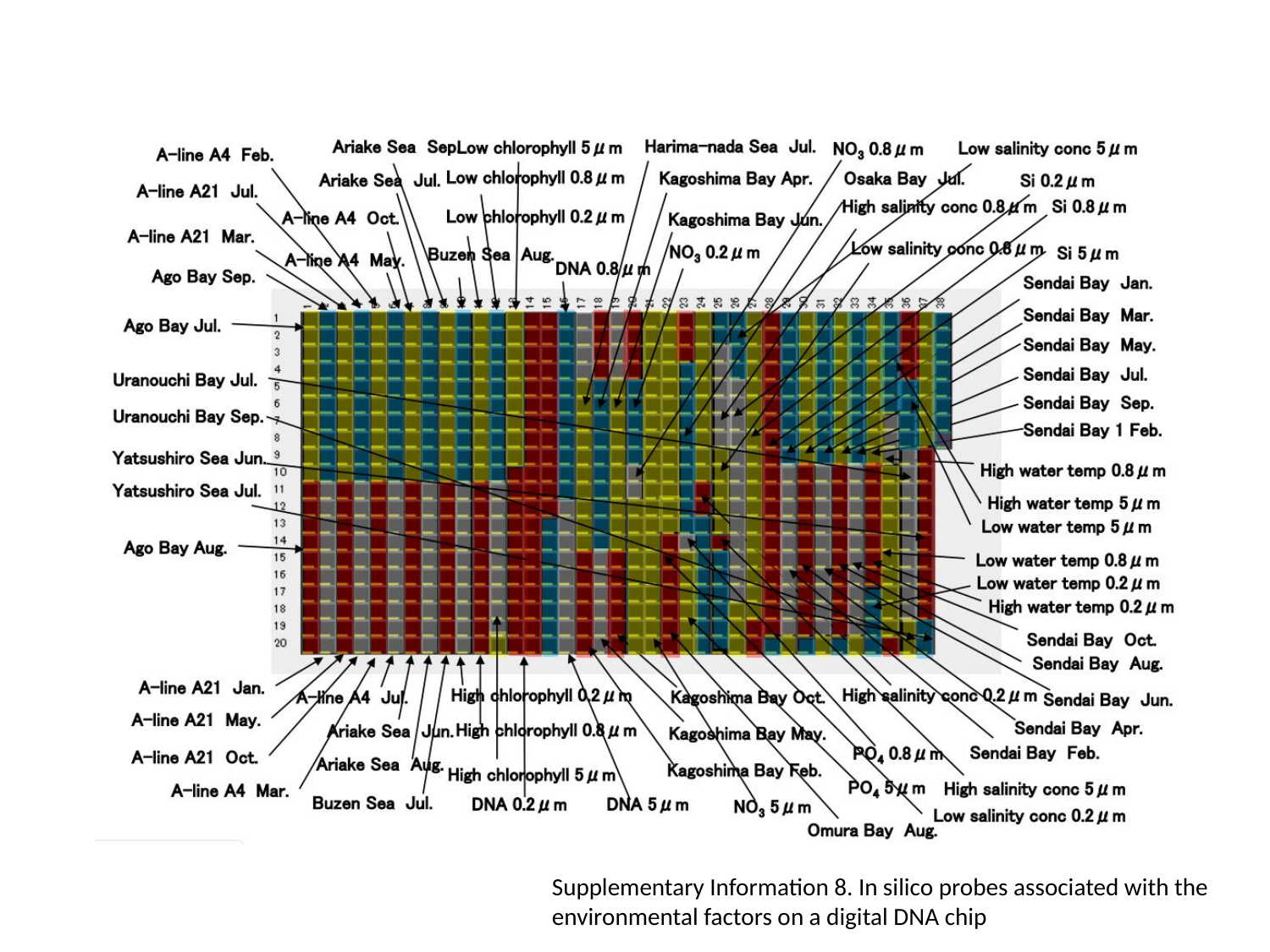

Supplementary Information 8. In silico probes associated with the environmental factors on a digital DNA chip
